# Flow behind an exponential shock wave in a rotational axisymmetric perfect gas with magnetic field and variable density

**DOI:** 10.1186/s40064-016-3119-z

**Published:** 2016-09-08

**Authors:** G. Nath, P. K. Sahu

**Affiliations:** Department of Mathematics, Motilal Nehru National Institute of Technology Allahabad, Allahabad, Uttar Pradesh 211004 India

**Keywords:** Self similar solution, Shock wave, Interstellar medium, Rotating medium, Adiabatic and Isothermal flows, Magnetogasdynamics

## Abstract

A self-similar model for one-dimensional unsteady isothermal and adiabatic flows behind a strong exponential shock wave driven out by a cylindrical piston moving with time according to an exponential law in an ideal gas in the presence of azimuthal magnetic field and variable density is discussed in a rotating atmosphere. The ambient medium is assumed to possess radial, axial and azimuthal component of fluid velocities. The initial density, the fluid velocities and magnetic field of the ambient medium are assumed to be varying with time according to an exponential law. The gas is taken to be non-viscous having infinite electrical conductivity. Solutions are obtained, in both the cases, when the flow between the shock and the piston is isothermal or adiabatic by taking into account the components of vorticity vector. The effects of the variation of the initial density index, adiabatic exponent of the gas and the Alfven-Mach number on the flow-field behind the shock wave are investigated. It is found that the presence of the magnetic field have decaying effects on the shock wave. Also, it is observed that the effect of an increase in the magnetic field strength is more impressive in the case of adiabatic flow than in the case of isothermal flow. The assumption of zero temperature gradient brings a profound change in the density, non-dimensional azimuthal and axial components of vorticity vector distributions in comparison to those in the case of adiabatic flow. A comparison is made between isothermal and adiabatic flows. It is obtained that an increase in the initial density variation index, adiabatic exponent and strength of the magnetic field decrease the shock strength.

## Background

The formulation of self-similar problems and examples describing adiabatic motion of non-rotating gas models of stars are discussed by Sedov (1959), Zel’Dovich and Raizer (1967), Lee and Chen (1968) and Summers (1975). The problem of propagation of magneto-gasdynamic shock waves in a rotating interplanetary atmosphere assumes special significance in the study of astrophysical phenomena. The experimental studies and astrophysical observations show that the outer atmosphere of the planets or stars rotates due to rotation of the planets or stars. Macroscopic motion with supersonic speed occurs in an interplanetary atmosphere with rotation and shock waves are generated. Further, the interplanetary magnetic field is connected with the rotation of the sun which implies that a large scale of magnetic field might appear in the rapidly rotating stars. Therefore, the rotation of planets or stars considerably affects the process happening in their outer layers, thus question connected with the explosions in rotating gas atmospheres are of definite astrophysical interest. Chaturani (1971) obtained the solutions for the propagation of cylindrical shock wave through a gas having solid body rotation by a similarity method adopted by Sakurai (1956). Nath et al. (1999) obtained the similarity solutions for the flow behind the spherical shock waves propagating in a non-uniform rotating interplanetary atmosphere with increasing energy. A theoretical model of propagation of strong spherical shock waves in a self-gravitating atmosphere with radiation flux in presence of a magnetic field and considering the medium behind the shock to be rotating but neglecting the rotation of the undisturbed medium was studied by Ganguly and Jana (1998). The self-similar solution for adiabatic flow headed by a magnetogasdynamic cylindrical shock wave in a rotating non-ideal gas is obtained by Vishwakarma et al. (2007).

Sedov (1959) (see Rao and Ramana 1976) indicated that a limiting case of a self-similar flow-field with a power-law shock is the flow-field formed with an exponential shock. Rao and Ramana (1976) obtained approximate analytical solutions for the problem of unsteady self-similar motion of a perfect gas displaced by a piston according to an exponential law.

The purpose of present work is to obtain the self-similar solutions for the flow behind the strong cylindrical shock wave generated by a moving piston in a rotational axisymmetric flow of a gas with variable density, variable azimuthal and axial fluid velocities under isothermal and adiabatic flow conditions (Levin and Skopina 2004; Nath 2010, 2011).

 Rao and Ramana (1976), Vishwakarma and Nath (2007) and Nath (2014, 2015) have studied the problem which we have considered in the present study by taking initial density constant without considering the effect of magnetic field in rotating or non-rotating medium. Singh et al. (2011) have considered same problem by taking initial magnetic field and initial density constant with the assumption that the gas to be non-ideal and medium to be non-rotating, whereas we have considered the medium to be rotating and the initial magnetic field and initial density decreasing exponentially. Shock waves through a variable-density medium have been treated by Sakurai (1956), Rogers (1957), Sedov (1959), Rosenau and Frankenthal (1976), Nath et al. (1999), Vishwakarma and Yadav (2003), Nath (2011) and others. Their results are more applicable to the shock formed in the deep interior of stars. Also, the material within star occurs within a strong magnetic field and the interplanetary magnetic field is connected with the rotation of the sun which implies that a large scale of magnetic field might appear in the rapidly rotating stars. Thus our problem is more realistic than the previous works corresponding to the physical phenomenon.

In the present work, therefore we investigate the one-dimensional unsteady self-similar rotational axisymmetric flow of a gas behind a strong shock driven out by a cylindrical piston moving with time according to an exponential law in the presence of magnetic field. It is assumed that the motion of the piston obeys the exponential law presented by Rao and Ramana (1976) (see also Vishwakarma and Nath 2006, 2007)

1 $$\begin{aligned} r_{p} = B \; exp(it),\quad i>0 , \end{aligned}$$

where $r_{p}$ is the radius of the piston, B and i are dimensional constants, and t is the time. ‘B’ represents the initial radius of the piston.

The law of piston motion (1) implies a boundary condition on the gas speed at the piston, which is required for the formulation of the problem. It is also assumed that the shock propagation follows the exponential law

2 $$\begin{aligned} r_{s}=\eta \;exp(it), \end{aligned}$$

where $r_{s}$ is the radius of the shock and $\eta$ is a dimensional constant which depends on the constant ‘B’ and the non-dimensional position of the piston [see Eq. (30)].

The analysis of the flow field in the region between the shock and the piston are presented for both the cases of adiabatic and isothermal flows. The isothermal flow assumption is physically realistic, when radiation heat transfer effects are implicitly present. The temperature behind the shock, as the shock propagates, increases and becomes very large so that there is intense transfer of energy by radiation and when intense heat exchange between particles of gas takes place, we may assume that there is no temperature gradient throughout the flow field, i.e., $\dfrac{\partial T}{\partial r}\rightarrow 0$ . Therefore, the temperature in the flow field depends only on time t and not on the distance r from the center of the explosion, i.e., $T=T(t)$, and the flow is isothermal as describe by Sedov (1959), Laumbach and Probstein (1970), Sachdev and Ashraf (1971) and Zhuravskaya and Levin (1996). This assumption on the character of the flow corresponds to the beginning of a very strong explosion (for example: underground, volcanic and cosmic explosions, coal-mine blasts) when the gas temperature is extremely high. A detailed mathematical theory of one-dimensional isothermal blast waves in a magnetic field was developed by Lerche (1979, 1981). With this assumption, we obtain the solutions in “Equations of motion and boundary conditions–isothermal flow” and “Self-similarity transformations” sections. In “Adiabatic flow” section, we present the solutions for the flow taken to be adiabatic.

The effects of variation of the Alfven-Mach number, the initial density variation index and the ratio of the specific heat of the gas on the shock strength and flow variables are investigated. It is found that the assumption of zero temperature gradient brings a profound change in the distribution of density, non-dimensional azimuthal and axial components of vorticity vectors as compared to those of the adiabatic case. A comparison between the obtained solutions and the existing solutions of Rao and Ramana (1976) is made in non-magnetic case. Also, a comparison between the solutions in the case of isothermal and adiabatic flows is made. Further, it is shown that the consideration of zero temperature gradient and an increase in the strength of ambient magnetic field, the initial density variation index or adiabatic exponent of the gas decrease the shock strength and widen the disturbed region between the shock and the piston. Effects of gravitation and viscosity are not taken into account.

## Equations of motion and boundary conditions–isothermal flow

In Eulerian coordinates, the system of equations of gas dynamics describing the unsteady, one-dimensional isothermal flow of rotational axisymmetric perfect gas under the influence of an azimuthal magnetic field, may be expressed in the form (c.f. Whitham 1958; Laumbach and Probstein 1970; Levin and Skopina 2004; Nath 2010, 2011)

3 $$\begin{aligned}&\dfrac{\partial \rho }{\partial t} + u \dfrac{\partial \rho }{\partial r} + \rho \dfrac{\partial u}{\partial r} + \dfrac{u \rho }{r} = 0, \end{aligned}$$

4 $$\begin{aligned}&\dfrac{\partial u}{\partial t} + u \dfrac{\partial u}{\partial r} + \dfrac{1}{\rho } \left[ \dfrac{\partial p}{\partial r} + \mu h \dfrac{\partial h}{\partial r} + \dfrac{\mu h^{2}}{r} \right] - \dfrac{v^{2} }{r} = 0, \end{aligned}$$

5 $$\begin{aligned}&\dfrac{\partial v}{\partial t} + u \dfrac{\partial v}{\partial r} + \dfrac{u v}{r} = 0, \end{aligned}$$

6 $$\begin{aligned}&\dfrac{\partial w}{\partial t} + u \dfrac{\partial w}{\partial r} = 0, \end{aligned}$$

7 $$\begin{aligned}&\dfrac{\partial h}{\partial t} + u \dfrac{\partial h}{\partial r} + h \dfrac{\partial u}{\partial r} = 0, \end{aligned}$$

8 $$\begin{aligned}&\dfrac{\partial T}{\partial r} = 0. \end{aligned}$$

where r and t are independent space and time coordinates; u, v, and w are the radial, azimuthal and axial components of the fluid velocity $\overrightarrow{q}$ in the cylindrical coordinates $(r,\theta ,z)$; *p*, $\rho$, *h* and *T* are the pressure, the density, the azimuthal magnetic field and the temperature; $\mu$ is the magnetic permeability. Here the electrical conductivity of the gas is assumed to be infinite.

Also, the relation between the angular velocity ‘A’ of the medium at radial distance r from the axis of symmetry and the azimuthal component of velocity is given by

9 $$\begin{aligned} v=Ar, \end{aligned}$$

The vorticity vector

$$\begin{aligned} \overrightarrow{\zeta }=\dfrac{1}{2} \; Curl \; \overrightarrow{q}, \end{aligned}$$

has the components

10 $$\begin{aligned} \zeta _{r} = 0,\quad \zeta _{\theta } = -\dfrac{1}{2} \dfrac{\partial w}{\partial r},\quad \zeta _{z} = \dfrac{1}{2 r} \dfrac{\partial }{\partial r} (rv). \end{aligned}$$

The system of Eqs. (3)–(8) should be supplemented with an equation of state. An ideal gas behaviour of the medium is assumed, so that (Nath 2011)

11 $$\begin{aligned} p = \rho RT, \end{aligned}$$

where *R* is the gas constant. The gas constant *R* and the temperature *T* are assumed to obey the thermodynamic relations $R = C_{p} - C_{v}$ and $e_{m} = C_{v} T$, where $C_{v} = \dfrac{R}{\gamma - 1}$ is the specific heat at constant volume and $e_{m}$ being the internal energy per unit mass of the gas can be written as

12 $$\begin{aligned} e_{m} = \dfrac{p}{(\gamma - 1) \rho }, \end{aligned}$$

where $\gamma$ is the ratio of specific heats.

A strong cylindrical shock wave is supposed to be propagating in the undisturbed ideal gas with variable density in the presence of an azimuthal magnetic field, which has zero radial velocity, variable azimuthal and axial velocities. Immediately ahead of the shock front, the flow variables are

13 $$\begin{aligned} u&= u_{a} = 0,\end{aligned}$$

14 $$\begin{aligned} \rho&= \rho _{a} = \rho _{0} \; exp(- \sigma t), \quad \sigma > 0 , \end{aligned}$$

15 $$\begin{aligned} v&= v_{a} = C \; exp(\delta t), \end{aligned}$$

16 $$\begin{aligned} w&= w_{a} = E \;exp(\alpha t), \end{aligned}$$

17 $$\begin{aligned} h&= h_{a} = h_{0} \; exp(- \lambda t), \end{aligned}$$

where $\rho _{0}$, *C*, *E*, $h_{0}$, $\sigma$, $\delta$, $\alpha$ and $\lambda$ are the dimensional constants, and the subscript ‘a’ refers to the conditions immediately ahead of the shock front.

Therefore, the components of the vorticity vector, ahead of the shock, vary as

18 $$\begin{aligned} \zeta _{r_{a}}&= 0, \end{aligned}$$

19 $$\begin{aligned} \zeta _{\theta _{a}}&= -\dfrac{E \alpha }{2 i r_{s}} \; exp(\alpha t),\end{aligned}$$

20 $$\begin{aligned} \zeta _{\theta _{a}}&= \dfrac{C ( i + \delta )}{2 i r_{s}} \;exp(\delta t). \end{aligned}$$

The initial angular velocity of the medium at radial distance $r_{s}$ is given by, from Eq. (9),

21 $$\begin{aligned} A_{a} = \dfrac{v_{a}}{r_{s}}. \end{aligned}$$

From Eqs. (21) and (15), we find that the initial angular velocity vary as

22 $$\begin{aligned} A_{a} = \dfrac{C \, exp(\delta t)}{r_{s}}. \end{aligned}$$

The jump conditions at the magnetogasdynamic shock wave are given by the conservation of mass, momentum and energy across the shock, namely,

23 $$\begin{aligned}&\rho _{a}V = \rho _{n} (V - u_{n}), \\&h_{a} V = h_{n} (V - u_{n}), \\&p_{a} + \dfrac{1}{2} \mu h_{a}^{2} + \rho _{a} V^{2} = p_{n} + \dfrac{1}{2} \mu h_{n}^{2} + \rho _{n} (V - u_{n})^{2}, \\&e_{m_{a}} + \dfrac{p_{a}}{\rho _{a}}+ \dfrac{1}{2} V^{2} + \dfrac{\mu h_{a}^{2}}{\rho _{a}} - \dfrac{F_{a}}{\rho _{a} V} = e_{m_{n}} + \dfrac{p_{n}}{\rho _{n}}+ \dfrac{1}{2} (V - u_{n})^{2} + \dfrac{\mu h_{n}^{2}}{\rho _{n}} - \dfrac{F_{n}}{\rho _{a} V}, \\&v_{a} = v_{n}, \\&w_{a} = w_{n}, \end{aligned}$$

where the subscript ‘n’ denotes the conditions immediately behind the shock front, $V \left( = \dfrac{dr_{s}}{dt}\right)$ denotes the velocity of the shock front and ‘F’ is the radiation heat flux. The pressure ahead of a strong shock is very small in comparison to the pressure behind of the shock, and therefore, it is neglected (Zel’Dovich and Raizer 1967)

24 $$\begin{aligned} p_{a} \approx 0, \quad e_{m_{a}} \approx 0. \end{aligned}$$

The shock conditions (23) across a strong shock propagating into an ideal gas reduce to

25 $$\begin{aligned} \rho _{n}&= \dfrac{\rho _{a}}{\beta }, \\ u_{n}&= (1 - \beta ) V, \\ p_{n}&= \left[ (1 - \beta ) + \frac{M_{A}^{-2}}{2} \left( 1 - \dfrac{1}{\beta ^{2}} \right) \right] \rho _{a} V^{2}, \\ v_{n}&= v_{a}, \\ w_{n}&= w_{a} , \\ h_{n}&= \dfrac{h_{a}}{\beta }, \end{aligned}$$

where $M_{A} = \left( \dfrac{\rho _{a} V^{2}}{\mu h_{a}^{2}}\right) ^{\frac{1}{2}}$ is the Alfven-Mach Number. The quantity $\beta \,(0< \beta < 1)$ is obtained by the quadratic relation

26 $$\begin{aligned} \beta ^{2}(\gamma + 1) - \beta \left[ \gamma \left( 1 + M_{A}^{-2}\right) - 1 \right] + (\gamma - 2) M_{A}^{-2} = 0, \end{aligned}$$

where $(F_{n} - F_{a})$ is neglected in comparison with the product of $p_{n}$ and *V* (Laumbach and Probstein 1970; Vishwakarma and Nath 2007; Nath 2011).

Equation (8) together with Eq. (11) gives

27 $$\begin{aligned} \dfrac{p}{p_{n}} = \dfrac{\rho }{\rho _{n}}. \end{aligned}$$

Following Levin and Skopina (2004) and Nath (2011), we obtained the jump conditions for the components of vorticity vector across the shock front as

28 $$\begin{aligned} \zeta _{\theta _{n}}&= \dfrac{\zeta _{\theta _{a}}}{\beta }, \\ \zeta _{z_{n}}&= \dfrac{\zeta _{z_{a}}}{\beta }. \end{aligned}$$

## Self-similarity transformations

Introducing $\xi = \dfrac{r}{r_{s}}$ as an independent variable, so that one may choose $\xi =1$ immediately behind the shock wave and $\xi = \xi _{p}$ at the piston face. The field variables describing the flow pattern can then be written in terms of the dimensionless functions of $\xi$ such that (Vishwakarma and Nath 2007; Singh et al. 2011; Nath 2014)

29 $$\begin{aligned} u&= V U(\xi ), \quad v = V \phi (\xi ), \quad w = V W(\xi ), \\ p&= \rho _{a} V^{2} P(\xi ), \quad \rho = \rho _{a} G(\xi ), \quad \sqrt{\mu }\, h = \sqrt{\rho _{a}}\; V H(\xi ), \end{aligned}$$

where *U*, $\phi$ , *W*, *P*, *G* and *H* are function of $\xi$ only.

A relation between *B* and $\eta$ can be obtained from Eqs. (1), (2) and (29) as

30 $$\begin{aligned} \eta = \dfrac{B}{\xi _{p}}. \end{aligned}$$

For the existence of similarity solutions ‘$M_{A}$’ should be constant, therefore

31 $$\begin{aligned} \sigma = 2 (i + \lambda ). \end{aligned}$$

Equations (29) and (27) gives a relation between P and G in the form

32 $$\begin{aligned} P(\xi ) = \left[ \beta (1 - \beta )+ \dfrac{ M_{A}^{-2} (\beta ^{2} - 1)}{2 \beta } \right] G(\xi ). \end{aligned}$$

Using the similarity transformations (29) and Eq. (32), we can transform the system of governing Eqs. (3)–(7) into the following system of ordinary differential equations:

33 $$\begin{aligned}&\dfrac{dU}{d\xi } + (U - \xi ) \dfrac{1}{G} \dfrac{dG}{d\xi } + \dfrac{U}{\xi }- \dfrac{\sigma }{i} = 0 , \end{aligned}$$

34 $$\begin{aligned}&\quad (U - \xi )\dfrac{dU}{d\xi } + \left[ \beta (1 - \beta )+ \dfrac{ M_{A}^{-2} (\beta ^{2} - 1)}{2 \beta } \right] \dfrac{1}{G} \dfrac{dG}{d\xi } + \dfrac{H}{G} \dfrac{dH}{d\xi } + \dfrac{H^{2}}{G \xi } + U - \dfrac{\phi ^{2}}{\xi } = 0 ,\end{aligned}$$

35 $$\begin{aligned}&\quad (U - \xi )\dfrac{d\phi }{d\xi } + \phi + \dfrac{U \phi }{\xi } = 0 , \end{aligned}$$

36 $$\begin{aligned}&\quad (U - \xi )\dfrac{dW}{d\xi } + W = 0 ,\end{aligned}$$

37 $$\begin{aligned}&\quad \dfrac{dU}{d\xi } + (U - \xi ) \dfrac{1}{H} \dfrac{dH}{d\xi } + \left( 1 - \dfrac{\sigma }{2i} \right) = 0 . \end{aligned}$$

Solving Eqs. (33)–(37) for $\dfrac{dU}{d\xi }$, $\dfrac{dG}{d\xi }$, $\dfrac{dH}{d\xi }$, $\dfrac{d\phi }{d\xi }$ and $\dfrac{dW}{d\xi }$, we have

38 $$\begin{aligned} \dfrac{dU}{d\xi }&= - \dfrac{(U - \xi )}{G} \, L \, \theta -\dfrac{U}{\xi } + \dfrac{\sigma }{i}, \end{aligned}$$

39 $$\begin{aligned} \dfrac{dG}{d\xi }&= L \, \theta , \end{aligned}$$

40 $$\begin{aligned} \dfrac{dH}{d\xi }&= \dfrac{H}{G} \, L \, \theta + \dfrac{H}{\xi } - \dfrac{\sigma H}{2i (U - \xi )} , \end{aligned}$$

41 $$\begin{aligned} \dfrac{d\phi }{d\xi }&= - \dfrac{\phi \, (U + \xi )}{\xi \, (U - \xi )} ,\end{aligned}$$

42 $$\begin{aligned} \dfrac{dW}{d\xi }&= - \dfrac{W}{(U - \xi )} , \end{aligned}$$

where

43 $$\begin{aligned} L = L(\xi ) = \left[ \dfrac{U^{2}}{\xi } - (U - \xi ) \dfrac{\sigma }{i} - \dfrac{2 H^{2}}{G \xi } + \dfrac{\sigma H^{2}}{ 2iG (U - \xi )} - 2 U + \dfrac{\phi ^{2}}{\xi } \right] , \end{aligned}$$

and

44 $$\begin{aligned} \theta = \theta (\xi ) = \dfrac{2 G^{2} \beta }{2G\beta ^{2} (1-\beta ) + G M_{A}^{-2} (\beta ^{2} - 1) + 2\beta H^{2} -2G\beta (U - \xi )^{2}}. \end{aligned}$$

Applying the similarity transformations on Eq. (9), we obtained the non-dimensional components of the vorticity vector $l_{r} = \dfrac{\zeta _{r}}{V/r_{s}}$, $l_{\theta }= \dfrac{\zeta _{\theta }}{V/r_{s}}$, $l_{z}= \dfrac{\zeta _{z}}{V/r_{s}}$ in the flow-filed behind the shock as

45 $$\begin{aligned} l_{r}&= 0,\end{aligned}$$

46 $$\begin{aligned} l_{\theta }&= \dfrac{W}{2 (U - \xi )},\end{aligned}$$

47 $$\begin{aligned} l_{z}&= - \dfrac{\phi }{(U - \xi )}. \end{aligned}$$

Using the shock conditions (25), the boundary conditions at the strong shock front are given by

48 $$\begin{aligned} G(1)&= \dfrac{1}{\beta } , \\ U(1)&= (1 - \beta ) , \\ P(1)&= \left[ (1 - \beta )+ \dfrac{M_{A}^{-2}}{2} \left( 1 - \dfrac{1}{\beta ^{2}} \right) \right] , \\ \phi (1)&= \dfrac{C}{i \eta }, \\ W(1)&= \dfrac{E}{i \eta }, \\ H(1)&= \dfrac{1}{\beta M_{A}} , \end{aligned}$$

where it is necessary to use $\delta = i = \alpha$ to obtain the similarity solution.

In addition to the shock conditions (48), the condition to be satisfied at the piston surface is that the velocity of the fluid is equal to the velocity of the piston itself. This kinematic condition at the piston face in non-dimensional form can be written as

49 $$\begin{aligned} U(\xi _{p}) = \xi _{p}. \end{aligned}$$

## Adiabatic flow

In this section, we present the self similar solution for the adiabatic flow behind a strong shock driven out by a cylindrical piston moving according to the exponential law (1), in the case of ideal gas with magnetic field. The strong shock conditions, which serve as the boundary conditions for the problem will be same as the shock conditions (25) in the case of isothermal flow.

For adiabatic flow, Eq. (8) is replaced by

50 $$\begin{aligned} \dfrac{\partial p}{\partial t} + u \dfrac{\partial p}{\partial r} - a^{2} \left( \dfrac{\partial \rho }{\partial t} + u \dfrac{\partial \rho }{\partial r} \right) = 0, \end{aligned}$$

where $a^{2} =\dfrac{\gamma p}{\rho }$ is the equilibrium speed of sound. Using Eqs. (11), (50) may be written as

51 $$\begin{aligned} \dfrac{\partial e_{m}}{\partial t} + u \dfrac{\partial e_{m}}{\partial r} - \dfrac{p}{\rho ^{2}} \left( \dfrac{\partial \rho }{\partial t} + u \dfrac{\partial \rho }{\partial r} \right) = 0. \end{aligned}$$

Using the similarity transformations (29), the system of governing Eqs. (3)–(7) and (51) can be transformed to the following system of ordinary differential equations:

52 $$\begin{aligned}&\dfrac{dU}{d\xi } + (U - \xi ) \dfrac{1}{G} \dfrac{dG}{d\xi } + \dfrac{U}{\xi }- \dfrac{\sigma }{i} = 0 , \end{aligned}$$

53 $$\begin{aligned}&\quad (U - \xi )\dfrac{dU}{d\xi } + \dfrac{1}{G} \left[ \dfrac{dP}{d\xi } + H \dfrac{dH}{d\xi } + \dfrac{H^{2}}{\xi } \right] + U - \dfrac{\phi ^{2}}{\xi } = 0 , \end{aligned}$$

54 $$\begin{aligned}&\quad (U - \xi )\dfrac{dP}{d\xi } - \dfrac{\gamma P}{G}(U - \xi ) \dfrac{dG}{d\xi } + 2P + (\gamma - 1) P \dfrac{\sigma }{i} = 0 , \end{aligned}$$

55 $$\begin{aligned}&\quad (U - \xi )\dfrac{d\phi }{d\xi } + \phi + \dfrac{\phi }{\xi } = 0 , \end{aligned}$$

56 $$\begin{aligned}&\quad (U - \xi )\dfrac{dW}{d\xi } + W = 0 ,\end{aligned}$$

57 $$\begin{aligned}&\quad \dfrac{dU}{d\xi } + (U - \xi ) \dfrac{1}{H} \dfrac{dH}{d\xi } + \left( 1 - \dfrac{\sigma }{2i} \right) = 0 . \end{aligned}$$

Solving Eqs. (52)–(57) for $\dfrac{dU}{d\xi }$, $\dfrac{dG}{d\xi }$, $\dfrac{dP}{d\xi }$, $\dfrac{dH}{d\xi }$, $\dfrac{d\phi }{d\xi }$ and $\dfrac{dW}{d\xi }$, we have

58 $$\begin{aligned} \dfrac{dU}{d\xi }&= - \dfrac{(U - \xi )}{\xi \left[ H^{2} + \gamma P - G (U - \xi )^{2} \right] } \, L' -\dfrac{U}{\xi } + \dfrac{\sigma }{i} , \end{aligned}$$

59 $$\begin{aligned} \dfrac{dG}{d\xi }&= \dfrac{G}{\xi \left[ H^{2} + \gamma P - G (U - \xi )^{2} \right] } \, L' , \end{aligned}$$

60 $$\begin{aligned} \dfrac{dP}{d\xi }&= \dfrac{\gamma P}{\xi \left[ H^{2} + \gamma P - G (U - \xi )^{2} \right] } \, L' -\dfrac{2P}{(U - \xi )}- \dfrac{ (\gamma - 1) P \sigma }{i (U - \xi ) } , \end{aligned}$$

61 $$\begin{aligned} \dfrac{dH}{d\xi }&= \dfrac{H}{\xi \left[ H^{2} + \gamma P - G (U - \xi )^{2} \right] } \, L' + \dfrac{H}{\xi }- \dfrac{\sigma H}{2i (U - \xi )} , \end{aligned}$$

62 $$\begin{aligned} \dfrac{d\phi }{d\xi }&= - \dfrac{\phi \, (U + \xi )}{\xi \, (U - \xi )} ,\end{aligned}$$

63 $$\begin{aligned} \dfrac{dW}{d\xi }&= - \dfrac{W}{(U - \xi )}, \end{aligned}$$

where

64 $$\begin{aligned} L' &= \dfrac{1}{(U - \xi )} \left[ (U - \xi )^{2}G \left( U - \dfrac{\xi \sigma }{i} \right) + 2 P \xi + \left\{ 2P (\gamma - 1)+ H^{2} \right\} \dfrac{ \xi \sigma }{2i} \right. \\&\left. - \left( 2 H^{2} + UG\xi -\phi ^{2} G \right) (U - \xi ) \right] . \end{aligned}$$

The shock conditions (25) take the form (48). In addition to the shock conditions (48), the kinematic condition at the piston surface (49) must be satisfied. Also, the non-dimensional component of the vorticity vector (45)–(47) will be same as in the case of isothermal flow.

Normalizing the variables $u, v, w, p, \rho$ and *h* with their respective values at the shock, we obtain

65 $$\begin{aligned} \dfrac{u}{u_{n}} &= \dfrac{U(\xi )}{U(1)} , \quad \dfrac{v}{v_{n}} = \dfrac{\phi (\xi )}{\phi (1)} , \quad \dfrac{w}{w_{n}} = \dfrac{W(\xi )}{W(1)},\\ \dfrac{p}{p_{n}}&= \dfrac{P(\xi )}{P(1)} , \quad \dfrac{\rho }{\rho _{n}} = \dfrac{G(\xi )}{G(1)}, \quad \dfrac{h}{h_{n}} =\dfrac{H(\xi )}{H(1)}. \end{aligned}$$

## Results and discussion

**Fig. 1:**
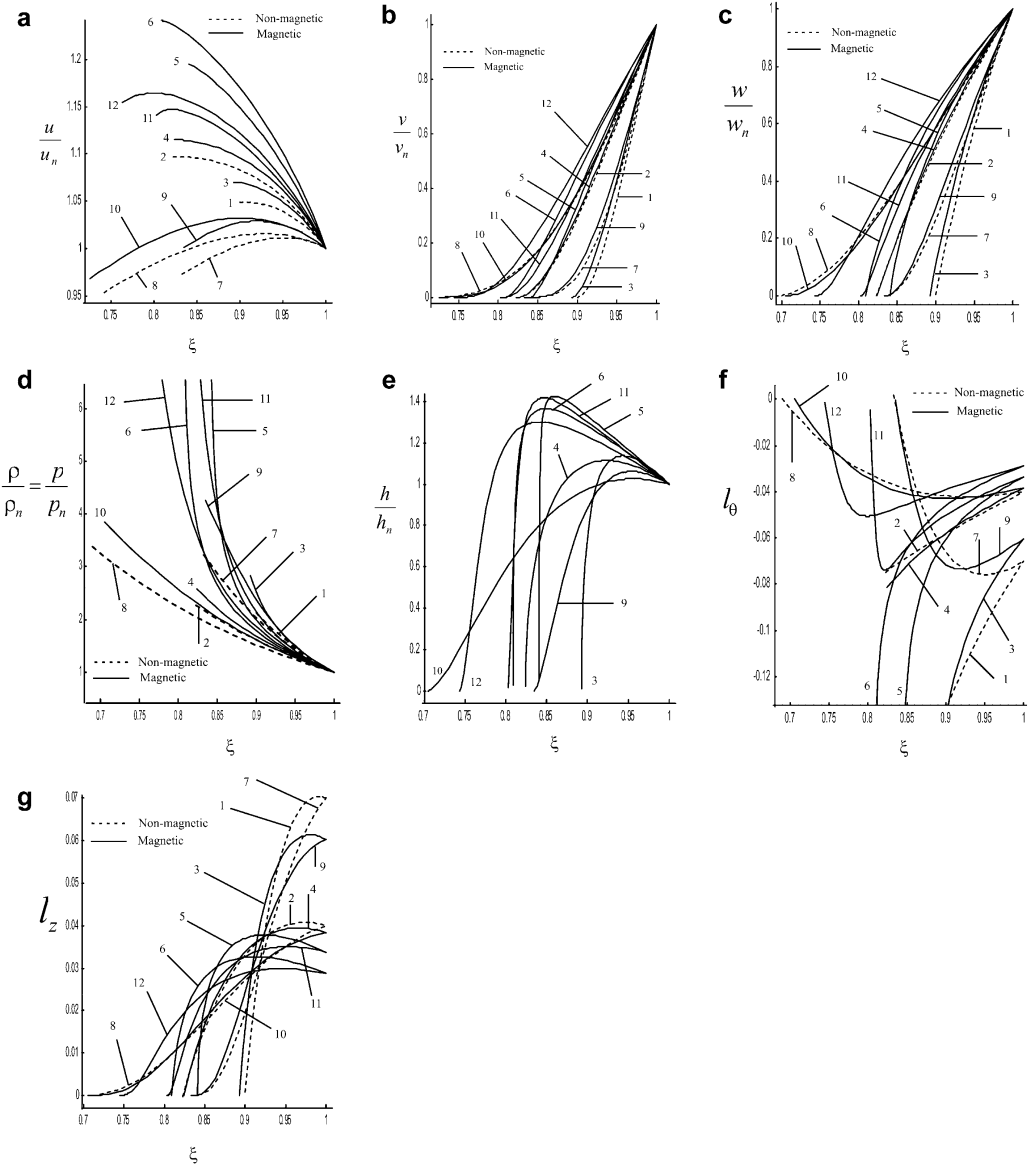
Variation of the reduced flow variables in the region behind the shock front in the case of isothermal flow: **a** radial component of fluid velocity $\dfrac{u}{u_{n}}$, **b** azimuthal component of fluid velocity $\dfrac{v}{v_{n}}$, **c** axial component of fluid velocity $\dfrac{w}{w_{n}}$, **d** density (pressure) $\dfrac{\rho }{\rho _{n}}$ $\left( =\dfrac{p}{p_{n}}\right)$, **e** azimuthal magnetic field $\dfrac{h}{h_{n}}$, **f** non-dimensional azimuthal component of vorticity vector $l_{\theta }$, **g** non-dimensional axial component of vorticity vector $l_{z}$: *1*. $M_{A}^{-2}=0$, $\gamma = \dfrac{4}{3}$, $\dfrac{\sigma }{i} =1$; *2*. $M_{A}^{-2} = 0$, $\gamma = \dfrac{5}{3}$, $\dfrac{\sigma }{i} =1$; *3*. $M_{A}^{-2} = 0.01$, $\gamma =\dfrac{4}{3}$, $\dfrac{\sigma }{i}=1$; *4*. $M_{A}^{-2}= 0.01$, $\gamma = \dfrac{5}{3}$, $\dfrac{\sigma }{i} =1$; *5*. $M_{A}^{-2} = 0.1$, $\gamma = \dfrac{4}{3}$, $\dfrac{\sigma }{i} =1$; *6*. $M_{A}^{-2}= 0.1$, $\gamma = \dfrac{5}{3}$, $\dfrac{\sigma }{i} =1$; *7*. $M_{A}^{-2} = 0$, $\gamma = \dfrac{4}{3}$, $\dfrac{\sigma }{i} =1.5$; *8*. $M_{A}^{-2} = 0$, $\gamma = \dfrac{5}{3}$, $\dfrac{\sigma }{i} =1.5$; *9*. $M_{A}^{-2} = 0.01$, $\gamma = \dfrac{4}{3}$, $\dfrac{\sigma }{i} =1.5$; *10*. $M_{A}^{-2} = 0.01$, $\gamma = \dfrac{5}{3}$, $\dfrac{\sigma }{i} =1.5$; *11*. $M_{A}^{-2} = 0.1$, $\gamma = \dfrac{4}{3}$, $\dfrac{\sigma }{i} =1.5$; *12*. $M_{A}^{-2}=0.1$, $\gamma = \dfrac{5}{3}$, $\dfrac{\sigma }{i} =1.5$

**Fig. 2:**
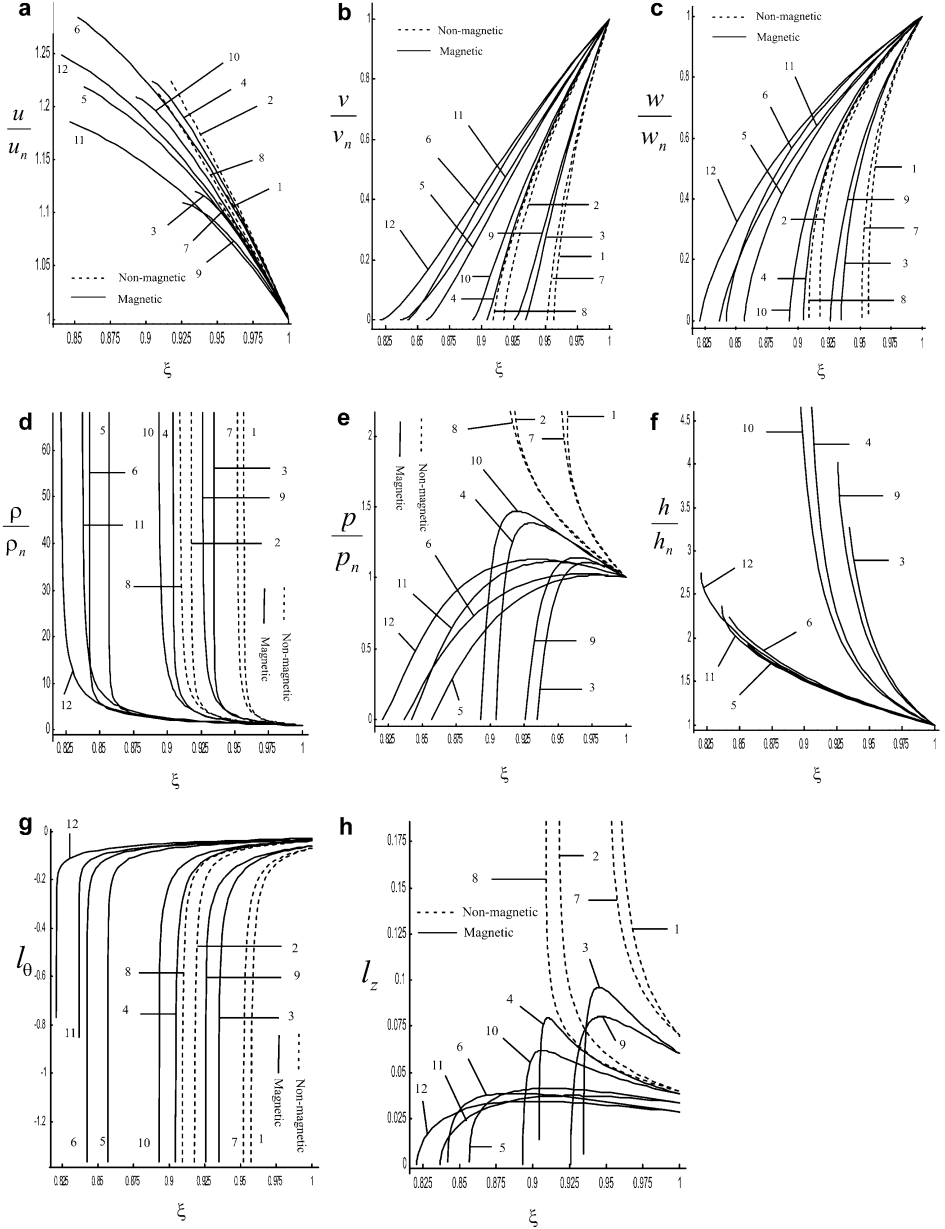
Variation of the reduced flow variables in the region behind the shock front in the case of adiabatic flow: **a** radial component of fluid velocity $\dfrac{u}{u_{n}}$, **b** azimuthal component of fluid velocity $\dfrac{v}{v_{n}}$, **c** axial component of fluid velocity $\dfrac{w}{w_{n}}$, **d** density $\dfrac{\rho }{\rho _{n}}$, **e** pressure $\dfrac{p}{p_{n}}$, **f** azimuthal magnetic field $\dfrac{h}{h_{n}}$, **g** non-dimensional azimuthal component of vorticity vector $l_{\theta }$, **h** non-dimensional axial component of vorticity vector $l_{z}$: *1*. $M_{A}^{-2} = 0$, $\gamma = \dfrac{4}{3}$, $\dfrac{\sigma }{i} =1$; *2*. $M_{A}^{-2} = 0$, $\gamma = \dfrac{5}{3}$, $\dfrac{\sigma }{i} =1$; *3*. $M_{A}^{-2} = 0.01$, $\gamma = \dfrac{4}{3}$, $\dfrac{\sigma }{i} =1$; *4*. $M_{A}^{-2} = 0.01$, $\gamma = \dfrac{5}{3}$, $\dfrac{\sigma }{i} =1$; *5*. $M_{A}^{-2} = 0.1$, $\gamma = \dfrac{4}{3}$, $\dfrac{\sigma }{i} =1$; *6*. $M_{A}^{-2} = 0.1$, $\gamma = \dfrac{5}{3}$, $\dfrac{\sigma }{i} =1$;    *7*. $M_{A}^{-2} = 0$, $\gamma = \dfrac{4}{3}$, $\dfrac{\sigma }{i} =1.5$;    *8*. $M_{A}^{-2} = 0$, $\gamma = \dfrac{5}{3}$ , $\dfrac{\sigma }{i} =1.5$; *9*. $M_{A}^{-2} = 0.01$, $\gamma = \dfrac{4}{3}$, $\dfrac{\sigma }{i} =1.5$; *10*. $M_{A}^{-2} = 0.01$, $\gamma = \dfrac{5}{3}$, $\dfrac{\sigma }{i} =1.5$; *11*. $M_{A}^{-2} = 0.1$, $\gamma = \dfrac{4}{3}$, $\dfrac{\sigma }{i} =1.5$; *12*. $M_{A}^{-2} = 0.1$, $\gamma = \dfrac{5}{3}$, $\dfrac{\sigma }{i} =1.5$

The distribution of the flow variables between the shock front $(\xi = 1)$ and the inner expanding surface or piston $(\xi = \xi _{p})$ is obtained by the numerical integration of Eqs. (38)–(42) for isothermal flow, and from Eqs. (58)–(63) for adiabatic flow with the boundary conditions (48) and (49) by the Runge–Kutta method of the fourth order. The values of the constant parameters, for the determination of numerical integration, are taken to be (Rosenau and Frankenthal 1976; Nath 2011) $\gamma = \dfrac{4}{3},\dfrac{5}{3} ; \; \dfrac{\sigma }{i} = 1, 1.5; \; M_{A}^{-2} = 0.0, 0.01, 0.1$. For fully ionized gas $\gamma = \dfrac{5}{3}$ and for relativistic gases $\gamma = \dfrac{4}{3}$, which are applicable to interstellar medium. These two values of $\gamma$ mark the most general range of values seen in real stars. For stars, the stability is related with the value of the adiabatic index in its interior that has to be larger than $\dfrac{4}{3}$ (Onsi et al. 1994; Casali and Menezes 2010). The stability of a star depends on the value of $\gamma$ in the core being larger than $\dfrac{4}{3}$, collapse beginning when $\gamma$ falls below $\dfrac{4}{3}$. However, as nuclear densities are approached in the core $\gamma$ will rise above $\dfrac{4}{3}$ again, with the result that the collapse will come rapidly to a halt, and be reversed into a bounce that may lead to a supernova explosion (Onsi et al. 1994). So, the above values of $\gamma$ are taken for calculations in the present problem. The above values of $M_{A}^{-2}$ are taken for calculations in the present problem because Rosenau and Frankenthal (1976) have shown that the effects of magnetic field on the flow-field behind the shock are significant when $M_{A}^{-2}\ge 0.01$. The non-magnetic case is represented by $M_{A}^{-2} =0$. In the present problem, we have taken initial density variation index $\dfrac{\sigma }{i} =1,1.5$ for numerical calculations i. e. initial density of the ambient medium is assumed to be decreasing. There is astrophysical evidence for the existence of shocks propagating in regions of variable density. In a stellar explosion, the shock wave is expected to accelerate through the outer stellar layers where the density is decreasing rapidly with height. A similar situation may occur for an explosion in the gaseous atmosphere of a galaxy. Self-similar solutions provide an excellent description of the shock propagation because the accelerating shock structure becomes independent of the nature of initial explosion (Chevalier 1990). The present work is the extension to the work of Rao and Ramana (1976) by taking into account the rotation of the medium and the azimuthal magnetic field with variable density (see Figs. 1b, c, e–g, 2b, c, f–h).

**Fig. 3:**
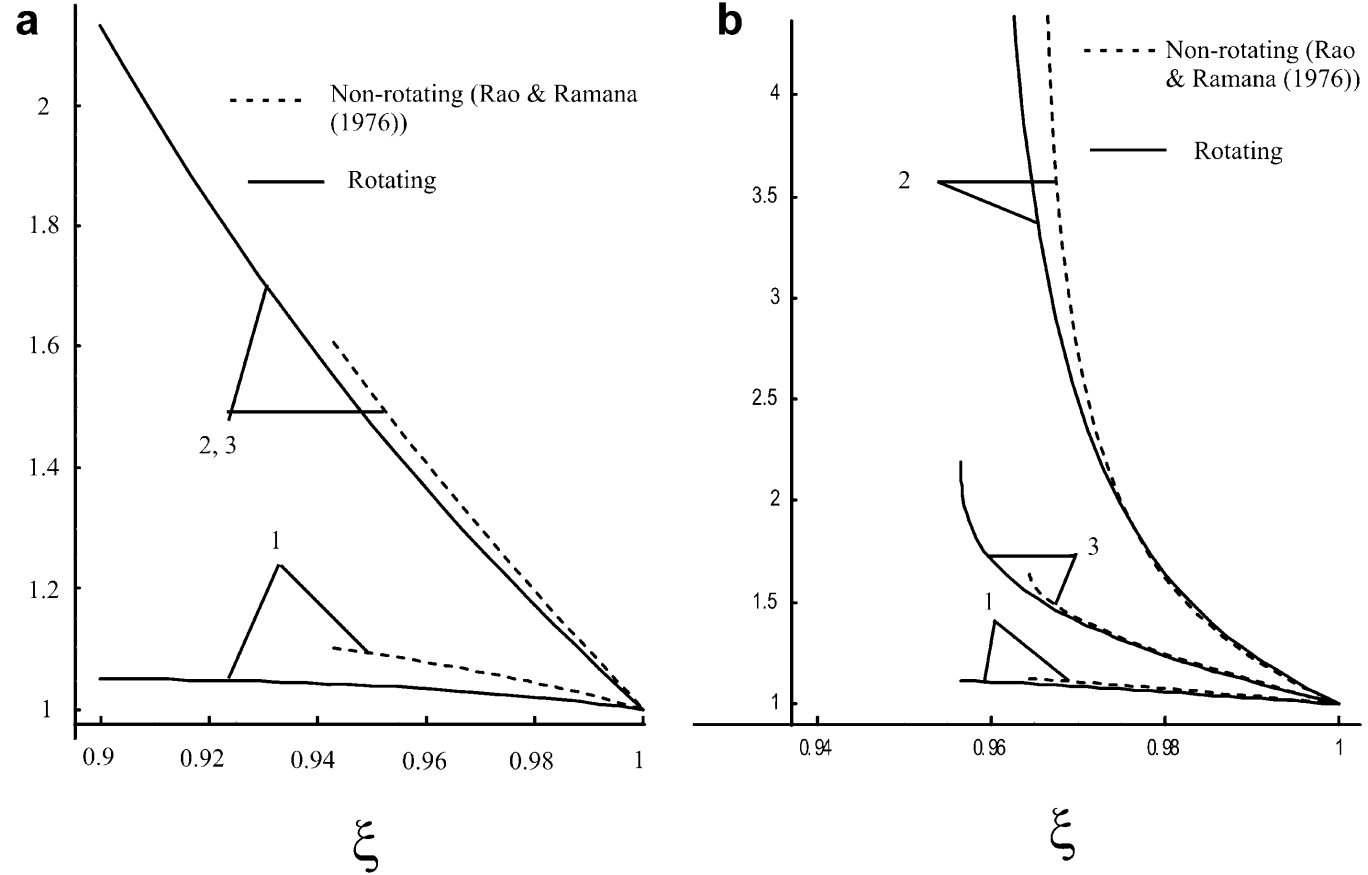
Variation of the reduced flow variables in the region behind the shock front for $M_{A}^{-2} =0$; $\gamma = \dfrac{4}{3}$; $\sigma = 0$ (non-rotating with constant density), $\dfrac{\sigma }{i} =1$ (rotating with variable density): **a** isothermal flow, **b** adiabatic flow: *1*. radial component of fluid velocity $\dfrac{u}{u_{n}}$, *2*. density $\dfrac{\rho }{\rho _{n}}$, *3*. pressure $\dfrac{p}{p_{n}}$

In non-magnetic case with constant density (i.e. $M_{A}^{-2} = 0$, $\rho _{a}$ = constant) our solution corresponds to the solution obtained by Rao and Ramana (1976) (Vishwakarma and Nath 2007 in the case of perfect gas for cylindrical symmetry i. e. $\overline{b} = 0$, $i = 1$; Vishwakarma and Nath 2006 in dust free case for cylindrical symmetry i. e. for $K_{p} = 0$, $i = 1$). To compare the obtained solution with the existing solution of Rao and Ramana (1976), the Fig. 3a, b are drawn in non-magnetic case. In Fig. 3a, b it is shown that the obtained solution is in good agreement with the existing solution of Rao and Ramana (1976). These figures demonstrate that the radial component of fluid velocity $\dfrac{u}{u_{n}}$, density $\dfrac{\rho }{\rho _{n}}$, pressure $\dfrac{p}{p_{n}}$ and the shock strength are decreasing for rotating medium than that in the case of non-rotating medium in the absence of magnetic field.

Table 1 shows the variation of density ratio $\beta \left( = \dfrac{\rho _{a}}{\rho _{n}} \right)$ across the shock front and the position of the piston $\xi _{p}$ for different values of $M_{A}^{-2}$, $\gamma$ and $\dfrac{\sigma }{i}$ in the isothermal and adiabatic cases.

**Table 1:** Variation of the density ratio $\beta \left( =\dfrac{\rho _{a}}{\rho _{n}}\right)$ across the shock front and the position of the piston surface $\xi _{p}$ for different values of $M_{A}^{-2}$, $\gamma$ and $\dfrac{\sigma }{i}$

$M_{A}^{-2}$	$\gamma$	$\beta$	Position of the piston surface $\xi _{p}$			
			Isothermal flow		Adiabatic flow	
			$\dfrac{\sigma }{i} = 1$	$\dfrac{\sigma }{i} = 1.5$	$\dfrac{\sigma }{i} = 1$	$\dfrac{\sigma }{i} = 1.5$
0	$\dfrac{4}{3}$	0.142857	0.899886	0.834074	0.956562	0.951338
	$\dfrac{5}{3}$	0.25000	0.822819	0.690086	0.917366	0.908795
0.01	$\dfrac{4}{3}$	0.165804	0.892594	0.835098	0.934578	0.925728
	$\dfrac{5}{3}$	0.261039	0.823930	0.705373	0.904250	0.892942
0.1	$\dfrac{4}{3}$	0.296396	0.840903	0.800793	0.856906	0.836709
	$\dfrac{5}{3}$	0.34838	0.808796	0.744513	0.842153	0.820695

Figures 1 and 2 show the variation of the flow variables $\dfrac{u}{u_{n}}, \dfrac{v}{v_{n}},\dfrac{w}{w_{n}} , \dfrac{\rho }{\rho _{n}}, \dfrac{p}{p_{n}} ,\dfrac{h}{h_{n}}$, the non-dimensional azimuthal component of vorticity vector $l_{\theta }$ and the non-dimensional axial component of vorticity vector $l_{z}$ against the similarity variable $\xi$ at various values of the parameters $M_{A}^{-2}$, $\gamma$ and $\dfrac{\sigma }{i}$ in the isothermal and adiabatic cases respectively.

Figures 1a–d and 2a–d show that the reduced radial component of fluid velocity $\dfrac{u}{u_{n}}$ and the reduced density $\dfrac{\rho }{\rho _{n}}$ increase; whereas the reduced azimuthal component of fluid velocity $\dfrac{v}{v_{n}}$ and the reduced axial component of fluid velocity $\dfrac{w}{w_{n}}$ decrease as we move from the shock front to the piston. Figures 1d and 2e show that the reduced pressure $\dfrac{p}{p_{n}}$ increases; but it decreases in the presence of magnetic field for adiabatic flow as we move from the shock front to the piston.

Figures 1e and 2f show that the reduced azimuthal magnetic field $\dfrac{h}{h_{n}}$ increases but in the case of isothermal flow it decreases after attaining the maximum value. Figures 1f and 2g show that the reduced azimuthal component of vorticity vector $l_{\theta }$ decreases; whereas it increases after attaining a minima for $\dfrac{\sigma }{i} = 1.5$ in case of isothermal flow.

From Table 1 and Figs. 1 and 2 it is found that the effects of an increase in the value of $M_{A}^{-2}$ (i.e. the effects of an increase in the strength of ambient magnetic field) are:

(i) to increase the value of $\beta$ i.e. to decrease the shock strength (see Table 1);
(ii) to decrease $\xi _{p}$ ingeneral (except the case when $\dfrac{\sigma }{i} = 1.5$, $\gamma = \dfrac{5}{3}$ for isothermal flow), i.e. to increase the distance of the piston from the shock front. Physically it means that the flow-field behind the shock become somewhat rarefied which is same as in (i) above (see Table 1);
(iii) the flow variables $\dfrac{u}{u_{n}}$, $\dfrac{\rho }{\rho _{n}}$, $\dfrac{p}{p_{n}}$ and $\dfrac{h}{h_{n}}$ increase in case of isothermal flow, but these flow variables decrease ingeneral in case of adiabatic flow (see Figs. 1a, d, e, 2a, d–f)
(iv) to increase the flow variables $\dfrac{v}{v_{n}}$ and $\dfrac{w}{w_{n}}$ ingeneral (see Figs. 1b, c, 2b, c);
(v) the non-dimensional azimuthal component of vorticity vector $l_{\theta }$ increases near shock and decreases near piston, but it increases at any point in the flow field behind the shock in the case of isothermal flow when $\dfrac{\sigma }{i} = 1$, $\gamma = \dfrac{4}{3}$; and in the case of adiabatic flow for all values of the parameters. (see Figs. 1f, 2g);
(vi) the non-dimensional axial component of vorticity vector $l_{z}$ decreases ingeneral; whereas it decreases near the shock and increases near the piston in the case of isothermal flow (see Figs. 1g, 2h).

It is found that the presence of magnetic field has decaying effect on shock wave. Also, it is observed that the effect of an increase in the magnetic field strength is more impressive in the case of adiabatic flow than in the case of isothermal flow (see Table 1). The density in the case of isothermal flow increases whereas decreases in the case of adiabatic flow with an increase in the strength of ambient magnetic field. Physically it means that gas compressed by shock wave moving perpendicular to the magnetic field will experience an increase in the field strength in direct proportion to increase in gas density in the case of isothermal flow whereas in the case of adiabatic flow gas compressed by shock wave will experience an increase in the field strength is inversely proportional to increase in gas density.

The effects of increasing value of adiabatic exponent of the gas $\gamma$ are

(i) the value of $\beta$ increased i.e. the shock strength is decreased (see Table 1);
(ii) the distance of the piston from the shock front is increased. This shows the same result as given in (i) above, i.e. there is a decrease in the shock strength (see Table 1);
(iii) to increase the flow variables $\dfrac{u}{u_{n}}$, $\dfrac{v}{v_{n}}$ and $\dfrac{w}{w_{n}}$, but to decrease the flow variables $\dfrac{\rho }{\rho _{n}}$ at any point in the flow-field behind the shock front (see Figs. 1a–d, 2a–d);
(iv) to decrease the reduced pressure $\dfrac{p}{p_{n}}$; whereas it increases in the case of adiabatic flow with magnetic field (i.e. $M_{A}^{-2}\ne 0$) (see Figs. 1d, 2e);
(v) to decrease the reduced azimuthal magnetic field $\dfrac{h}{h_{n}}$ for $M_{A}^{-2}=0.01$ and to increase it for $M_{A}^{-2} = 0.1$ in case of adiabatic flow; whereas it decreases near shock and increases near piston in case of isothermal flow (see Figs. 1e, 2f);
(vi) to increase the non-dimensional azimuthal component of vorticity vector $l_{\theta }$; whereas in the case of isothermal flow it increases near shock and decreases near piston when $\dfrac{\sigma }{i} = 1.5$ (see Figs. 1f, 2g);
(vii) to decrease the non-dimensional axial component of vorticity vector $l_{z}$ near shock and increase near piston; but it decreases at any point in the flow field behind the shock in the case of adiabatic flow, when $M_{A}^{-2} = 0$ (see Figs. 1g, 2h).

It is found that the increase in adiabatic exponent of gas has decaying effect on shock wave. Also, it is observed that an increase in the strength of the ambient magnetic field or the adiabatic exponent of the gas have similar effects on the azimuthal and axial components of fluid velocity with initial density variation index $\dfrac{\sigma }{i} = 1$ . Also, it is observed that the effect of an increase in adiabatic exponent of the gas is more impressive in the case of isothermal flow than in the case of adiabatic flow (see Table 1).

The effects of increasing value of initial density variation index $\dfrac{\sigma }{i}$ are

(i) to decrease $\xi _{p}$ i.e. to decrease the shock strength (see Table 1);
(ii) to decrease the flow variables $\dfrac{u}{u_{n}}$, but to increase the flow variables $\dfrac{v}{v_{n}}$, $\dfrac{w}{w_{n}}$ and $l_{\theta }$ at any point in the flow-field behind the shock front (see Figs. 1a–c, f, 2a–c, g;
(iii) to decrease $\dfrac{\rho }{\rho _{n}}$; whereas in the case of isothermal flow it increases near shock and decreases near piston when $M_{A}^{-2}=0.1$ (see Figs. 1d, 2d);
(iv) to increase $\dfrac{p}{p_{n}}$ when $M_{A}^{-2} \ne 0$ for adiabatic flow and when $M_{A}^{-2} = 0.1$ for isothermal flow; whereas it decreases when $M_{A}^{-2}= 0, 0.01$ for isothermal flow and when $M_{A}^{-2} = 0$ for adibatic flow (see Figs. 1d, 2e);
(v) to decrease $\dfrac{h}{h_{n}}$ in the case of adiabatic flow, but in the case of isothermal flow it decreases near shock and increases near piston (see Figs. 1e, 2f);
(vi) to decrease $l_{z}$ near shock and increases near piston, but in the case of adiabatic flow it decreases at any point in the flow field in the absence of magnetic field (i.e. when $M_{A}^{-2} = 0$) (see Figs. 1g, 2h).

It is found that the increase in value of initial density variation index has decaying effect on shock wave. Also, it is observed that the effect of increasing initial density variation index is more impressive in the case of isothermal flow than in the case of adiabatic flow (see Table 1).

## Conclusion

The present work investigates the self-similar flow behind a strong exponential cylindrical shock wave, propagating in a rotational axisymmetric ideal gas in the presence of azimuthal magnetic field for isothermal and adiabatic flows. The shock wave is driven out by a piston moving with time according to an exponential law. The article concerns with the explosion problem in rotating conducting medium, however the methodology and analysis presented here may be used to describe many other physical systems involving non-linear hyperbolic partial differential equations. The shock waves in rotational axisymmetric perfect gas with decreasing initial density and magnetic field can be important for description of shocks in supernova explosions, in the study of a flare produced shock in solar wind, central part of star burst galaxies, nuclear explosion, rupture of a pressurized vessel etc. On the basis of this work, one may draw the subsequent conclusions:

(i) The distance between shock and piston increases (i.e. shock strength decreases) with an increase in the strength of the ambient magnetic field $M_{A}^{-2}$, the adiabatic exponent $\gamma$ or the initial density variation index $\dfrac{\sigma }{i}$.
(ii) An increase in the value of $\dfrac{\sigma }{i}$ decrease the flow variables $\dfrac{u}{u_{n}}$, $\dfrac{\rho }{\rho _{n}}$, $\dfrac{h}{h_{n}}$, $l_{z}$ ingeneral; whereas in the case of the flow variables $\dfrac{v}{v_{n}}$, $\dfrac{w}{w_{n}}$ and $l_{\theta }$ the reverse behaviour is observed.
(iii) An increase in the value of initial density variation index $\dfrac{\sigma }{i}$ or adiabatic exponent of the gas $\gamma$ have same behaviour on the flow variables $\dfrac{v}{v_{n}} , \dfrac{w}{w_{n}} , l_{z}$ and the shock strength; whereas these parameters have opposite behaviour on the flow variable $\dfrac{u}{u_{n}}$. Also, by increasing the value of $\dfrac{\sigma }{i}$ or $\gamma$ the flow variables $\dfrac{\rho }{\rho _{n}}$, $\dfrac{p}{p_{n}}$ and $\dfrac{h}{h_{n}}$ show the same behaviour (except the cases when $M_{A}^{-2}= 0.1$ ).
(iv) An increase in $\gamma$ or $M_{A}^{-2}$ increases the radial velocity $\dfrac{u}{u_{n}}$ in case of isothermal flow; whereas in the case of adiabatic flow it decreases with an increase in $M_{A}^{-2}$ and increases with an increase in $\gamma$. Also, the flow variables $\dfrac{v}{v_{n}}$ and $\dfrac{w}{w_{n}}$ increase with an increase in $M_{A}^{-2}$ or $\gamma$, when $\dfrac{\sigma }{i} = 1$.
(v) The novel applications of this study include analysis of data from exploding wire experiments in conducting medium and cylindrically symmetric hypersonic flow problems associated with meteors or reentry vehicles (Hutchens 1995). Also, the solutions obtained can be used to interpret measurements carried out by space craft in the solar wind and in neighbourhood of the Earths magnetosphere.

Thus, it is found that presence of magnetic field, an increase in the value of initial density variation index or adiabatic index of the gas have decaying effect on shock wave. The distribution of the flow variables in the region between the shock and the piston are presented for both the cases of adiabatic and isothermal flows. The consideration of zero temperature gradient decreases the shock strength and widens the disturbed region between the shock and the piston. It is found that the assumption of zero temperature gradient brings a profound change in the distribution of density, non-dimensional azimuthal and axial components of vorticity vectors as compared to those of the adiabatic case and the other flow variable are little affected. Also, consideration of zero temperature gradient removes the singularities in the density, the non-dimensional axial and azimuthal components of vorticity vector near the piston ingeneral which arise in the case of the adiabatic flow.
